# Polymorphism of terthio­phene with surface confinement

**DOI:** 10.1107/S2052252518003949

**Published:** 2018-03-29

**Authors:** Roland Resel, Andrew O. F. Jones, Guillaume Schweicher, Roland Fischer, Nicola Demitri, Yves Henri Geerts

**Affiliations:** a Institut für Festkörperphysik, Technische Universität Graz, Petersgasse 16, Graz 8010, Austria; b Laboratoire de Chimie des Polymères, Faculté des Sciences, Université Libre de Bruxelles, Campus de la Plaine, Bruxelles 1050, Belgium; c Optoelectronics Group, Cavendish Laboratory, University of Cambridge, J. J. Thomson Avenue, Cambridge CB3 0HE, UK; d Institut für Anorganische Chemie, Technische Universität Graz, Stremayrgasse 9, Graz 8010, Austria; e Elettra-Sincrotrone, S. S. 14 Km 163.5 in Area Science Park, Basovizza, Trieste 34149, Italy

**Keywords:** molecular crystals, surface-mediated polymorphism, thermal gradient crystallization, single-crystalline films, specular X-ray diffraction

## Abstract

The origin of unknown polymorphic phases within thin films is still not well understood. This work reports on crystals of the molecule terthio­phene which were grown by thermal gradient crystallization using glass-plate substrates. The crystalline domains displayed a plate-like morphology with an extended lateral size of about 100 µm, but a thickness of only a few µm. Specular X-ray diffraction patterns confirmed the presence of a new polymorph of terthio­phene. Crystal structure solution from a single crystal peeled from the film revealed a structure with an extremely large unit-cell volume containing 42 independent molecules. In contrast to the previously determined crystal structure of terthio­phene, a herringbone packing motif was observed where the terminal ends of the molecules are arranged within one plane (*i.e.* the molecular packing conforms to the flat substrate surface). This type of molecular packing is obtained by 180° flipped molecules combined with partially random (disordered) occupation. A densely packed interface between terthio­phene crystallites and the substrate surface is obtained, this confirms that the new packing motif has adapted to the flat substrate surface.

## Abstract 2

The appearance of a new polymorphic phase is observed for terthio­phene as a result of adaptation to a flat substrate surface present during the crystallization process. This new polymorph can be attributed to the confinement of the molecular packing within the plane of the substrate surface accompanied by a tremendous increase in the unit-cell size and partial molecular disorder.

## 1. Introduction   

The crystallization of molecular materials at solid surfaces is often associated with the appearance of previously unknown polymorphic phases. Such substrate-induced (or thin-film) phases are frequently observed for conjugated molecules used as organic semiconductors, but examples from pharmaceutical molecules have also been shown in recent work (Reischl *et al.*, 2015 ▸; Jones *et al.*, 2016 ▸). The properties of these phases, especially the origins of their appearance, are not clear, so several questions relating to these phases arise: are these phases metastable and how can these phases be transformed to more thermodynamically stable phases? (Gundlach *et al.*, 1999 ▸; Gbabode *et al.*, 2012 ▸; Jones *et al.*, 2015 ▸; Truger *et al.*, 2016 ▸). Which growth kinetics cause new phases and what is the role of the substrate in the formation of new polymorphs? (Wedl *et al.*, 2012 ▸; Chung & Diao, 2016 ▸). A further open question is related to the influence of the substrate surface properties (*e.g.* surface energy, roughness *etc*.) on the formation of new polymorphs (Hiszpanski *et al.*, 2017 ▸). A common observation related to these phases is that the topography of a substrate surface induces a specific arrangement of molecules, therefore new molecular arrangements act as nuclei for the growth of new polymorphs at the interface with the substrate surface (Jones *et al.*, 2016 ▸). This can be discussed in terms of a topographic adaptation of molecular crystals to the surface, similar to the arrangement of the crystalline lattices observed for the epitaxial growth of organic molecules at surfaces (Wittmann & Lotz, 1990 ▸). The simplest case would be an atomically flat substrate where the molecular packing has to arrange along a terminal plane towards the substrate surface (*i.e.* no ‘gaps’ are present in the molecular packing at the interface). Proof of such topographic adaptation can be obtained by high-quality crystal structure solution of a substrate-induced phase. However, this is difficult to perform for such systems, since the size of the crystallites is often limited to the sub µm regime. Crystal structure solution from thin films would, in theory, be a reasonable tool to determine the molecular packing relative to the substrate surface (Schiefer *et al.*, 2007 ▸; Krauss *et al.*, 2008 ▸). However, the limited number of available reflections obtained from thin-film diffraction patterns (*e.g.* from grazing-incidence diffraction experiments) does not allow sufficient accuracy in the determination of the molecular conformation and the arrangements within surface-induced crystal structures (Yoshida *et al.*, 2007 ▸; Mannsfeld *et al.*, 2011 ▸).

In this work, we have selected the molecule 2,2′:5′,2′′-terthiophene (3T) for crystallization at a glass surface. The molecule is known to form plate-like crystals that are grown from an ether solution or by sublimation (Bolhuis *et al.*, 1989 ▸; Azumi *et al.*, 2003 ▸). A crystal structure has been determined previously by single-crystal X-ray diffraction; the compound crystallizes in a layered herringbone structure in the space group *P*2_1_/*c* with lattice constants *a* = 15.225 (4), *b* = 5.635 (3), *c* = 25.848 (3) Å and *β* = 98.15 (2)° at a temperature of 130 K (Bolhuis *et al.*, 1989 ▸). The asymmetric unit consists of two molecules and there is a total of eight molecules in the unit cell (*Z* = 8). Despite two different methods of crystal growth used in previous reports, their structures are identical (Azumi *et al.*, 2003 ▸). Another polymorph of 3T has been observed by crystallization from the melt using thermal gradient crystallization; however, the crystal structure could not be unambiguously solved (Schweicher *et al.*, 2011 ▸). In this work, we have isolated the new polymorph as a high-temperature phase by optimization of the growth conditions and present its structure. The 3T crystallites were a sufficient size so that the crystal structure could be solved by single-crystal X-ray diffraction. This polymorph shows a molecular packing motif which reveals one possible reason for the appearance of a substrate-induced phase.

## 2. Methods   

**Figure 1:**
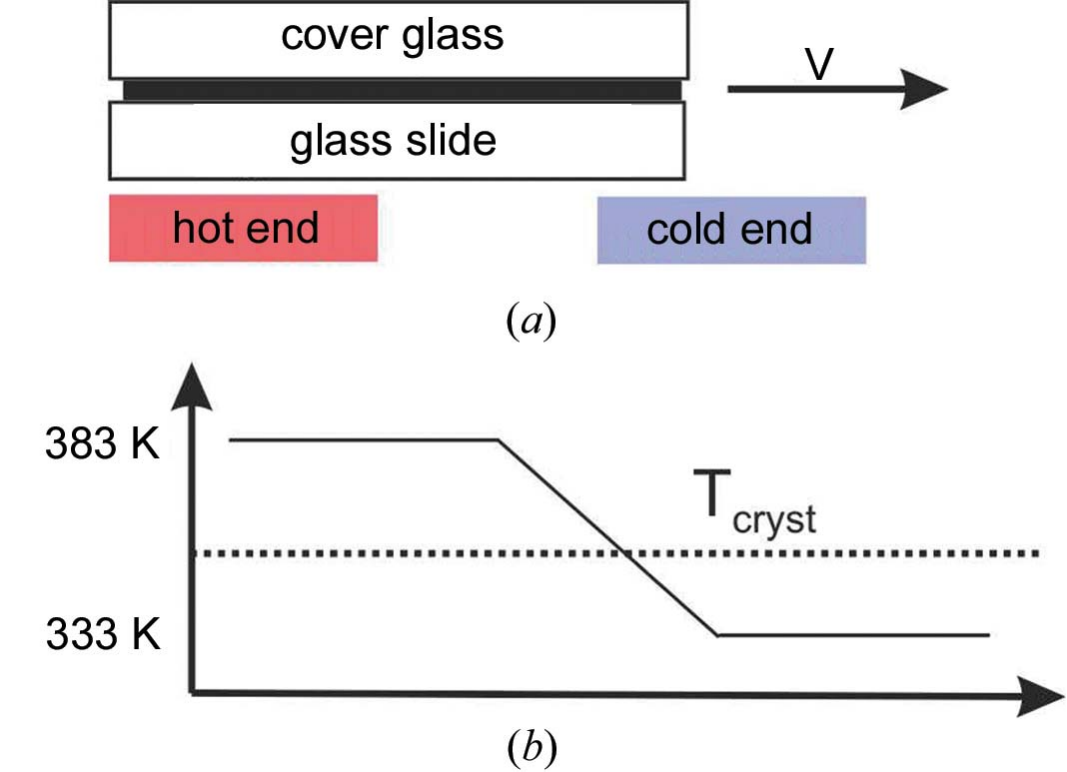
(*a*) Schematic picture of the gradient crystallization set-up where a layer of terthiophene squeezed between two glass plates is drawn with the velocity *v* across a temperature gradient. (*b*) The temperature distribution across the set-up with the crystallization temperature of terthiophene (*T*_cryst_) located between the two heated zones.

The material 2,2′:5′,2′′-terthio­phene was purchased from Aldrich with a purity of 99% and was used without further purification. The samples were prepared by thermal gradient crystallization; a schematic drawing of this crystallization technique is given in Fig. 1 ▸. A precise description of the set-up used for thermal gradient crystallization is reported elsewhere (Schweicher *et al.*, 2011 ▸). Terthio­phene, in a quantity of 3.5 mg, was squeezed between a cleaned D263 Borosilicate thin glass slide (Präzisions Glas & Optik GmbH – 10 × 16 × 0.7 mm) and a cleaned D263 Borosilicate cover glass Cat. No. 0101040 (Marienfeld – 10 × 16 × 0.16 mm), so that a crystal thickness ranging between 20 and 50 µm can be expected. The hot and the cold end of the thermal gradient set-up was adjusted to temperatures of 383 and 333 K, respectively. Crystallization from the molten state was obtained by moving the sample with a withdrawal velocity of 5 µm s^−1^ from the hot end towards the cold end. In contrast to previous crystallization methods of 3T (Schweicher *et al.*, 2011 ▸), the crystallization process was repeated twice; the sample was turned by 180° after the first transit and the aligned end of the sample was used as a seed for the second pass. After the crystallization process, the two substrates were separated. The morphology of the crystallites prepared at the substrate surface was investigated with a Nikon Eclipse 80i optical microscope equipped with a Nikon DS-5M digital camera.

Specular X-ray diffraction measurements were performed with a Philips X’Pert system using Cr *K*α radiation (λ = 2.2910 Å) in combination with a secondary graphite monochromator. For the single-crystal structure analysis, individual crystals were removed from the substrate by an adhesive Kapton tape or by gentle scratching of the surface. Diffraction data from the single crystals were collected from two different sources. First, a Bruker D8 Kappa diffractometer equipped with a SMART APEX II CCD detector using Mo *K*α radiation (λ = 0.71073 Å) from a microsource at a temperature of 100 K. Then, the solutions for the crystal structures were obtained by direct methods and structural refinement performed using *SHELXS*97 (Sheldrick, 2008 ▸). The space-group assignments and structural solutions were evaluated using *PLATON* (Spek, 2003 ▸). Second, the film was characterized at 100 and 293 K, scraping single-crystal fragments from the surface, using XRD from a high-brilliance synchrotron source. Data collections were performed at the X-ray diffraction beamline (XRD1) of the Elettra Synchrotron, Trieste (Italy) (Lausi *et al.*, 2015 ▸), with a Pilatus 2M hybrid-pixel area detector. Complete data sets were collected with a monochromatic wavelength of 0.700 Å through the rotating crystal method. The 3T fragments scraped from the surface were dipped in *N*-paratone and mounted onto the goniometer head with a nylon loop. The diffraction data were indexed and integrated using *XDS* (Kabsch, 2010 ▸). A semi-empirical absorption correction and scaling was performed, exploiting multiple measurements of symmetry-related reflections, using the program *SADABS* (Sheldrick, 2012 ▸). The structure was solved by the dual space algorithm implemented in the *SHELXT* code (Sheldrick, 2015*a* ▸). Fourier analysis and refinement were performed by full-matrix least-squares based on *F* ^2^ implemented in *SHELXL*2016/6 (Sheldrick, 2015*b* ▸). The program *Coot* was used for modelling (Emsley *et al.*, 2010 ▸). Anisotropic thermal motion was then applied to all atoms with an occupancy greater than 50%. Extensive disorder has been found for nine of the eleven 3T molecules found in the asymmetric unit (ASU), therefore extensive geometric and thermal motion parameter restraints (DFIX, FLAT, SADI, DELU and SIMU) have been applied to all fragments with partial occupancies. Hydrogen atoms were included at calculated positions with isotropic *U*_factors_ = 1.2*U*_eq_ (*U*_eq_ being the equivalent isotropic thermal factor of the bonded non-hydrogen atom). The same monoclinic crystalline form has been found at room and cryogenic temperatures (with a volume contraction of 3.9% upon cooling). A close-packed structure with no residual electron density has been found. Essential crystal and refinement data (Table 1 ▸) are reported.

## 3. Results and discussion   

**Figure 2:**
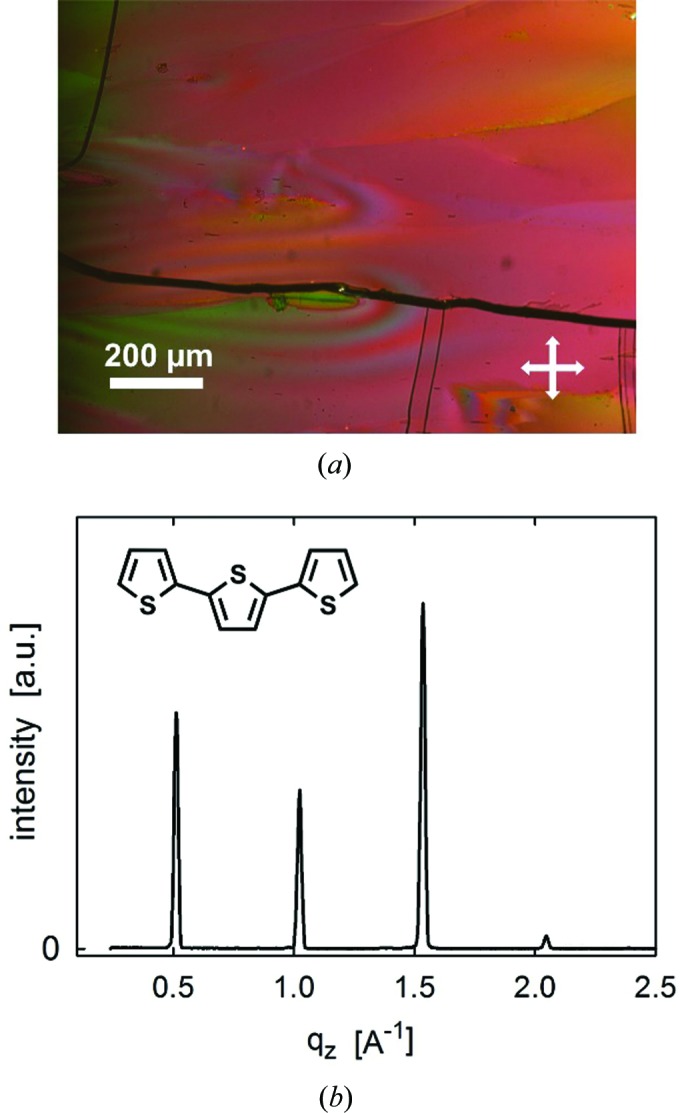
(*a*) Optical microscopy image using crossed polarizers and (*b*) specular X-ray diffraction pattern of terthio­phene crystals prepared by thermal gradient crystallization on a glass substrate. The inset provides the chemical structure of the molecule terthiophene.

The morphology of the crystals was investigated by optical microscopy using transmitted polarized light (Fig. 2 ▸ *a*). A plate-like morphology with lateral extensions of several hundred micrometres and a constant thickness has been observed. The single-crystal domains are separated by cracks which appear during cooling to room temperature as a result of the different thermal expansion coefficients of the organic material and the inorganic substrate. The homogeneous alignment of the crystallites is spread over the entire substrate, homogeneity is observed even at the starting point of the crystallization process at the substrate edge. Specular X-ray diffraction measurements reveal a Bragg peak at *q_z_* = 0.512 Å^−1^ and its higher order reflections at *q_z_* = 1.024 and 1.536 Å^−1^ (Fig. 2 ▸ *b*). These peaks arise from lattice planes with an interplanar distance of *d* = 12.27 Å. This distance cannot be assigned to the known crystal structure of 3T, but arises from the high-temperature phase (here denoted as the new phase) where the structure has not been solved completely (Schweicher *et al.*, 2011 ▸).

**Figure 3:**
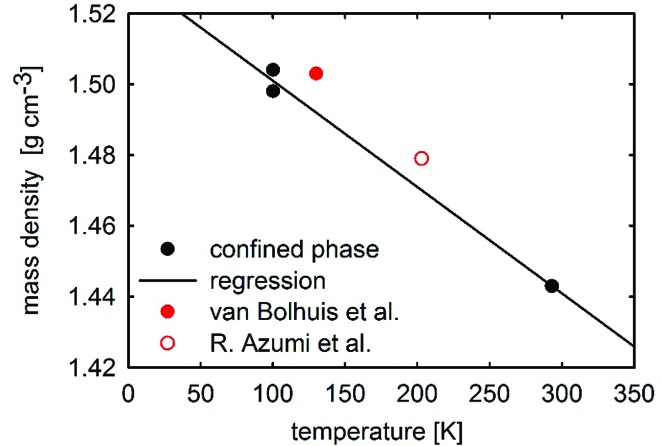
Calculated mass densities of the terthiophene phases as a function of temperature. The previously determined phase is represented by the red circles (van Bolhuis *et al.*, 1989 ▸; Azumi *et al.*, 2003 ▸), the surface-confined phase of this work is indicated by black circles.

Single-crystal structure solution of this phase using the XRD1 beamline at the Elettra synchrotron source at 100 K reveals a herringbone packing of the 3T molecules, as is often found for rod-like conjugated molecules (Desiraju & Gavezzotti, 1989 ▸). Crystallographic information for the single-crystal structure solution is listed in Table 1 ▸. A monoclinic crystal structure in the *P*2_1_/*n* space group is found. The asymmetric unit contains 10.5 molecules, so that there are in total 42 molecules in the unit cell with a cell volume of 11 536 Å^3^ and a mass density of 1.502 g cm^−3^. A second crystal was investigated by in-house X-ray diffraction experiments at the same temperature, revealing an identical molecular packing motif, though with a slightly larger unit-cell volume (11 563 Å^3^) and a slightly reduced mass density of 1.498 g cm^−3^. Measurements were also performed using synchrotron radiation at room temperature (293 K); here, the molecular packing does not change, but the unit-cell dimensions expand to *a* = 44.038 (9), *b* = 5.769 (1), *c* = 49.069 (10) Å, *β* = 105.59 (3)°, with a volume of 12 008 (4) Å^3^ and a mass density of 1.443 g cm^−3^. The mass densities are plotted as a function of temperature in Fig. 3 ▸; the straight line gives an interpolation of the measured mass densities of both investigated crystals at a temperature of 100 K and of the single-crystal solution at 293 K. Additionally, the mass densities of the known polymorphs of 3T are given at the respective temperatures in Fig. 3 ▸. It should be noted that the crystal structure solution in Bolhius *et al.* (1989) shows a comparable mass density (1.503 g cm^−3^ at 130 K) to the phase reported here. However, consideration of the thermal expansion of the unit-cell volume (represented by the straight line in Fig. 3 ▸) reveals that at comparable temperatures, a less dense molecular packing would be present in the new phase than in the previously reported phase. This means that, according to the mass density rule, the new polymorph is probably metastable and not the most thermodynamically stable phase (Burger & Ramberger, 1979 ▸).

**Figure 4:**
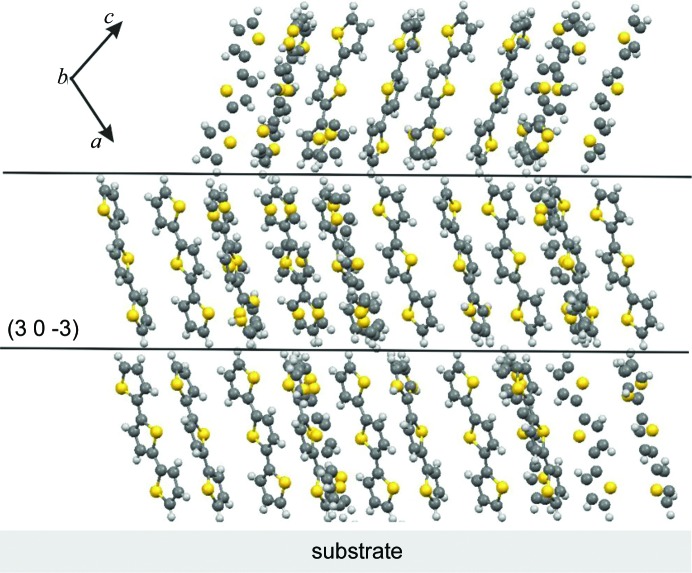
Arrangement of terthiophene molecules relative to the substrate surface within the surface-confined phase in a front view projected along the *b* axis. The crystallographic plane (30−3) is arranged parallel to the substrate surface.

Next, we discuss the molecular packing within the new polymorph. The molecules are arranged in layers in which three distinct layer types are found that vary by the tilt angles of the molecules towards different sides (Fig. 4 ▸). After a sequence of three layers, the stacking of the layers continues periodically. Within a single layer the molecules are packed with a herringbone motif, so that the aromatic planes of the neighbouring molecules are tilted by about 55° relative to each other. However, not all molecules are arranged in the same manner within the herringbone pattern, some molecules are considerably disordered meaning that a *cis* conformation and a 180° molecular flip is possible. These disarrangements are clearly visible in Fig. 4 ▸. In contrast, the known crystal structure of 3T shows only molecules in the *trans* conformation, and no 180° flipped or disordered molecules are present. There, a herringbone layer is formed by identically aligned molecules enclosing a herringbone angle of 59° between neighbouring molecules.

However, the most striking difference between the two polymorphs of 3T is that the phase presented here shows a planar boundary of the herringbone layers. This means that the terminal ends of the molecules are arranged at one topographical level so that a flat plane confines a single herringbone layer. Therefore, we can designate this polymorph as a confined phase. In the case of the previously known structure of 3T, the confinement of molecules is only observed in the crystallographic *b* direction. Whereas for the new polymorph, the confinement is found in two dimensions, *i.e.* the herringbone layers are able to adapt to the two-dimensional substrate surface. Note that the packing for both polymorphs along the *b* axis is practically identical, which results in nearly equal lattice constants in that direction.

The crystallographic planes with the Miller indices (30−3) are parallel to the herringbone layers, their interplanar distances (*d*) of 12.29 Å are calculated based on the single-crystal solution. This value is in excellent agreement with the observed distances from the specular X-ray diffraction pattern (Fig. 2 ▸ *b*). Since specular X-ray diffraction probes only crystallographic planes that are parallel to the substrate surface, it can be concluded that the herringbone layers of the crystals within our samples are oriented parallel to the substrate surfaces. The two experimental observations of (i) confined herringbone layers which are (ii) oriented parallel to the substrate surface, reveal that the presence of a surface is crucial for the polymorph formation.

It is well known that the surfaces of herringbone layers are typically low energy surfaces and these surfaces are responsible for the plate-like morphology (Nabok *et al.*, 2008 ▸). However, the origin of confined herringbone layers is less clear. One mechanism would be a further reduction of surface energies due to maximizing intermolecular interactions at the surface (and also within the bulk), so that at elevated temperatures a rearrangement of the confined herringbone layers towards the previously known 3T phase could be possible (Schweicher *et al.*, 2011 ▸). Another mechanism is related to interaction energies at the interface between a substrate surface and 3T molecules. Dense packing of the molecules with the substrate surface results in an energy gain, so that an adaptation to the surface by the molecules would result in confinement with the substrate surface. In summary, the crystallization of the polymorph is associated with frustration between competing solid-state synthons which, for this system, are represented by the efficient molecular packing due to quadrupolar intermolecular interactions and flat surface constraints, used for crystal growth. As a consequence, a high number of molecules (*Z* = 10.5) are part of the asymmetric unit (Anderson *et al.*, 2008 ▸).

## 4. Conclusions   

A new polymorph of terthiophene (3T) has been prepared by thermal gradient crystallization and the crystal structure was solved using single-crystal X-ray diffraction. The similarities between the new polymorph and the previously documented phase of 3T are the formation of herringbone layers and the stacking of these layers upon each other. The molecular packing within one herringbone layer along the *b* axis is identical for both structures, where the terminal ends of the molecules complete the herringbone layer at the same level. However, the new polymorph presented here shows confinement in two dimensions, which is achieved by 180° flipped molecules and randomly reversed molecules at specific molecular sites. This molecular rearrangement is associated with a considerable enlargement of the crystallographic unit cell, which contains 42 molecules. Since the mass density of the new polymorph is slightly smaller than the previously documented phase, a metastable character of this polymorph can be concluded. The driving mechanism for the confinement cannot be given, however, because of the specific crystallization technique used here, an adaptation of the molecular packing to a flat substrate surface seems to play an important role and further highlights the potential for surfaces to be used to tune crystal packing.

## Supplementary Material

Crystal structure: contains datablock(s) I. DOI: 10.1107/S2052252518003949/ro5010sup1.cif

CCDC reference: 1552752

## Figures and Tables

**Table 1:** Crystallographic information of the surface-confined polymorph of terthiophene

	3T Polymorph II
CCDC number	1552752
Chemical formula	C_12_H_8_S_3_
Formula weight (g mol^−1^)	248.37
Temperature (K)	100 (2)
Wavelength (Å)	0.700
Crystal system	Monoclinic
Space group	*P*2_1_ */n*
Unit-cell dimensions (Å, °)	*a* = 43.822 (9), *b* = 5.689 (1), *c* = 48.331 (10), *α* = 90, *β* = 106.79 (3), *γ* = 90
Volume (Å^3^)	11536 (4)
*Z*	42
Density (calculated) (g cm^−3^)	1.502
Absorption coefficient (mm^−1^)	0.598
*F*(000)	5376
Crystal size (mm)	0.10 × 0.10 × 0.01
Crystal habit	Colourless thin plates
Resolution (Å)	0.74
θ range for data collection (°)	0.54–28.23
Index ranges	−59 ≤ *h* ≤ 59 −7 ≤ *k* ≤ 7 −65 ≤ *l* ≤ 65
Reflections collected	127933
Independent reflections [data with *I* > 2σ(*I*)]	29739 (20951)
Data multiplicity (max resolution)	4.15 (4.07)
*I*/σ(*I*) (max. resolution)	11.23 (6.27)
*R*_merge_ (max. resolution)	0.0464 (0.1448)
Data completeness (max. resolution) (%)	99.7 (99.1)
Refinement method	Full-matrix least-squares on *F* ^2^
Data/restraints/parameters	29739/512/1903
Goodness-of-fit on *F* ^2^	1.029
Δ/σ_max_	0.023
Final *R* indices [*I* > 2σ(*I*)]†	*R*_1_ = 0.0631, w*R*_2_ = 0.1687
*R* indices (all data)†	*R*_1_ = 0.0908, w*R*_2_ = 0.1909
Largest difference peak and hole (e Å^−3^)	1.583 and −1.3650
R.m.s. deviation from mean (e Å^−3^)	0.085

† *R*_1_ = ∑||*F*_o_| – |*F*_c_||/∑|*F*_o_|, *wR*_2_ = {∑[*w*(*F*_o_^2^ – *F*_c_^2^)^2^]/∑[*w*(*F*_o_^2^)^2^]}^1/2^.
